# Physical Vapor Transport Growth of Antiferromagnetic CrCl_3_ Flakes Down to Monolayer Thickness

**DOI:** 10.1002/advs.202203548

**Published:** 2022-12-01

**Authors:** Jia Wang, Zahra Ahmadi, David Lujan, Jeongheon Choe, Takashi Taniguchi, Kenji Watanabe, Xiaoqin Li, Jeffrey E. Shield, Xia Hong

**Affiliations:** ^1^ Department of Physics and Astronomy & Nebraska Center for Materials and Nanoscience University of Nebraska‐Lincoln Lincoln NE 68588‐0299 USA; ^2^ Department of Mechanical and Materials Engineering University of Nebraska‐Lincoln Lincoln NE 68588‐2526 USA; ^3^ Department of Physics University of Texas at Austin Austin TX 78712‐1192 USA; ^4^ International Center for Materials Nanoarchitectonics National Institute for Materials Science 1‐1 Namiki Tsukuba Ibaraki 305‐0044 Japan; ^5^ Research Center for Functional Materials National Institute for Materials Science 1‐1 Namiki Tsukuba Ibaraki 305‐0044 Japan

**Keywords:** CrCl_3_, physical vapor transport, tunnel junction, tunneling magnetoresistance, van der Waals magnet

## Abstract

The van der Waals magnets CrX_3_ (X = I, Br, and Cl) exhibit highly tunable magnetic properties and are promising candidates for developing novel two‐dimensional (2D) spintronic devices such as magnetic tunnel junctions and spin tunneling transistors. Previous studies of the antiferromagnetic CrCl_3_ have mainly focused on mechanically exfoliated samples. Controlled synthesis of high quality atomically thin flakes is critical for their technological implementation but has not been achieved to date. This work reports the growth of large CrCl_3_ flakes down to monolayer thickness via the physical vapor transport technique. Both isolated flakes with well‐defined facets and long stripe samples with the trilayer portion exceeding 60 µm have been obtained. High‐resolution transmission electron microscopy studies show that the CrCl_3_ flakes are single crystalline in the monoclinic structure, consistent with the Raman results. The room temperature stability of the CrCl_3_ flakes decreases with decreasing thickness. The tunneling magnetoresistance of graphite/CrCl_3_/graphite tunnel junctions confirms that few‐layer CrCl_3_ possesses in‐plane magnetic anisotropy and Néel temperature of 17 K. This study paves the path for developing CrCl_3_‐based scalable 2D spintronic applications.

## Graphical abstract

Large van der Waals antiferromagnet CrCl_3_ flakes down to monolayer thickness have been synthesized by the physical vapor transport technique. The samples have the monoclinic structure with high crystallinity and homogeneous stoichiometry. Tunneling magnetoresistance of graphite/CrCl_3_/graphite tunnel junctions reveals in‐plane magnetic anisotropy and *T*_N_ of 17 K for few‐layer CrCl_3_. This work paves the path for developing CrCl_3_‐based scalable 2D spintronics.

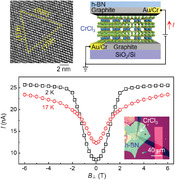

## 1 Introduction

Since their discovery, two‐dimensional (2D) van der Waals (vdW) magnets CrX_3_ (X = Cl, Br, I) have attracted extensive research interests for their unusual magnetic properties^[^ ^1^, ^2^, ^3^, ^4^, ^5^, ^6^, ^7^, ^8^, ^9^, ^10^ ^]^ compared with conventional magnetic metals and oxides.^[^ ^11^, ^12^, ^13^ ^]^ They are flexible, can sustain the magnetic ground state down to monolayer thickness,^[^ ^6^, ^7^, ^9^, ^10^ ^]^ and can be stacked with other vdW materials to create multifunctional heterostructures.^[^ ^1^, ^2^, ^3^, ^4^, ^5^, ^6^, ^14^, ^15^, ^16^, ^17^, ^18^ ^]^ It has been shown that the magnetic order and magnetic anisotropy of CrX_3_ can be sensitively tuned by strain and doping,^[^ ^16^, ^17^, ^18^, ^19^ ^]^ making it a versatile playground for studying magnetic quantum phase transitions and designing novel energy‐efficient spintronic devices, including magnetic tunnel junctions,^[^ ^1^, ^2^, ^3^, ^4^, ^5^ ^]^ spin tunneling field–effect transistors,^[^ ^16^, ^17^, ^18^ ^]^ and quantum spin Hall systems.^[^ ^15^ ^]^ CrCl_3_ is an A‐type antiferromagnet with in‐plane magnetic anisotropy.^[^ ^1^, ^2^, ^3^, ^4^ ^]^ Previous studies have mainly focused on mechanically exfoliated samples.^[^ ^1^, ^2^, ^3^, ^4^, ^5^, ^8^, ^20^, ^21^ ^]^ While nanosheets of CrCl_3_ have been deposited via the chemical vapor transport (CVT) method, only samples thicker than 25 nm have been obtained.^[^ ^22^ ^]^ Controlled synthesis of high‐quality atomically thin flakes is of great fundamental and technological interests but has not been achieved to date.

In this work, we report the direct growth of large CrCl_3_ flakes down to monolayer thickness via the physical vapor transport (PVT) technique. Triangular and hexagonal thin flakes with well‐defined facets as well as long stripe samples with the trilayer portion exceeding 60 µm have been obtained. High‐resolution transmission electron microscopy (HRTEM) studies show that the CrCl_3_ flakes are single crystalline with the monoclinic structure, consistent with the Raman characterizations. The sample stoichiometry has been confirmed by scanning electron microscopy (SEM)‐energy dispersive X‐ray spectroscopy (EDS) studies. Atomic force microscopy (AFM) studies show that the room temperature stability of CrCl_3_ flakes decreases with decreasing thickness. Characterization of graphite/CrCl_3_/graphite tunneling devices reveals a Néel temperature (*T*_N_) of 17 K and in‐plane magnetic anisotropy in few‐layer CrCl_3_. Our study enables scalable synthesis of high‐quality atomically thin CrCl_3_ flakes, paving the path for their implementation in 2D spintronic applications.

## 2 Results and Discussion

### 2.1 Synthesis of CrCl_3_ Flakes

**Figure 1:**
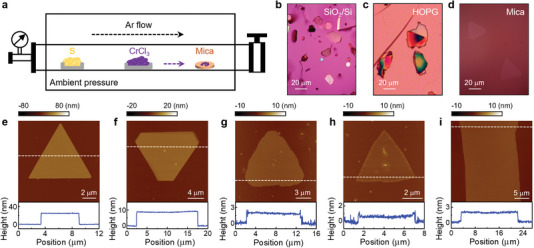
Synthesis of thick to monolayer CrCl_3_ flakes. a) Schematic of the experimental setup for PVT growth of CrCl_3_. b‐d) Optical images of as‐grown CrCl_3_ flakes on b) SiO_2_/Si, c) HOPG, and d) mica substrates. e–i) AFM images of CrCl_3_ flakes on mica with different thicknesses (upper panels), with the height profiles along the dashed lines (lower panels). The averaged flake thicknesses are 24.8 ± 0.2 nm, 9.25 ± 0.04 nm, 1.84 ± 0.01 nm (trilayer), 0.63 ± 0.07 nm (monolayer), and 1.9 ± 0.1 nm (trilayer), respectively.

2D vdW CrCl_3_ flakes are synthesized from CrCl_3_ powder using the PVT technique (**Figure** **1**a, see Experimental Section for growth details). The samples are deposited on three types of substrates: mica (fluorophlogopite, [KMg_3_(AlSi_3_O_10_)F_2_]), highly oriented pyrolytic graphite (HOPG), and SiO_2_/Si substrates. We then investigate the effects of substrates on the lateral size, flake thickness, and crystalline orientation of the samples. As shown in Figure 1b, CrCl_3_ on SiO_2_/Si prefers vertical growth and forms relatively thick crystals. Horizontal growth of large size thin flakes has been achieved on HOPG (Figure 1c) and mica (Figure 1d) substrates, which can be attributed to their atomically smooth and dangling‐bond‐free surfaces. Previous studies have shown that such surfaces can effectively promote the attachment of precursors on the layer edges and facilitate subsequent horizontal growth.^[^ ^23^, ^24^ ^]^ As the flakes deposited on HOPG do not have well‐defined facets (Figure 1c) and are hard to isolate from the underneath graphite pieces, we next focus on characterizing the samples deposited on mica.

We have obtained both isolated flakes with triangular and hexagonal shapes and long stripe samples on mica. Figure 1e–i shows the AFM topography images of five CrCl_3_ samples with different thicknesses. The flakes thicker than 9 nm show well‐defined facets with sharp edges (Figure 1e,f). The few‐layer to monolayer CrCl_3_ flakes (Figure 1g,h) also possess the triangular shape, but the edges are rough with micro‐facets and the corners are rounded. This has been attributed to the CrCl_3_ desorption during growth. For ultrathin flakes, there is an insufficient growth time for the edge atoms to reach thermodynamic equilibrium.^[^ ^25^ ^]^ In previous studies, CVT‐grown CrCl_3_ nanosheets are mostly thicker than 25 nm,^[^ ^22^ ^]^ and ultrathin flakes have only been obtained via mechanical exfoliation.^[^ ^1^, ^2^, ^3^, ^4^, ^5^, ^20^ ^]^ Our study is the first report of direct growth of monolayer CrCl_3_ (Figure 1h). Systematic AFM imaging on a large scale reveals over 25% yield of ultrathin flakes, including monolayer, bilayer, and trilayer samples (Figure S1a,b, Supporting Information). In addition to the isolated flakes, we have also achieved long stripes of ultrathin CrCl_3_ samples. Figure 1i shows the trilayer portion (66 µm by 20 µm) of a long stripe sample, whose overall length is over 1 mm (Figure S1c, Supporting Information). The ultrathin portion of the stripe samples can exceed 60% (Section S1, Supporting Information).

We examine the room temperature stability of the CrCl_3_ flakes by taking a series of AFM images with time after growth.^[^ ^20^, ^26^ ^]^ It has been shown that CrCl_3_ is more stable compared with CrI_3_.^[^ ^20^, ^21^, ^26^ ^]^ For a 64 nm flake, there is no obvious change in the sample morphology for about 5 months (Figure S2a, Supporting Information), showing excellent room temperature stability. The thinner flakes, on the other hand, show clear degradation with time. The 20 nm flake remains stable on Day 23, while bubble‐like features emerge on the sample surface on Day 37 (Figure S2b, Supporting Information). Similar degradation signs have been reported in exfoliated CrCl_3_ flakes,^[^ ^20^, ^26^ ^]^ which is attributed to the formation of CrCl_2_.^[^ ^26^ ^]^ For the monolayer flakes, the sample surface becomes rough on Day 6 (Figure S2c, Supporting Information), with the flake thickness increasing from 0.69 to 3.05 nm (Figure S2d, Supporting Information). It is possible that the sample degradation has started even before it becomes discernable in AFM measurements, as previously reported in Cr_2_Ge_2_Te_6_.^[^ ^27^ ^]^

### 2.2 Sample Characterization

**Figure 2:**
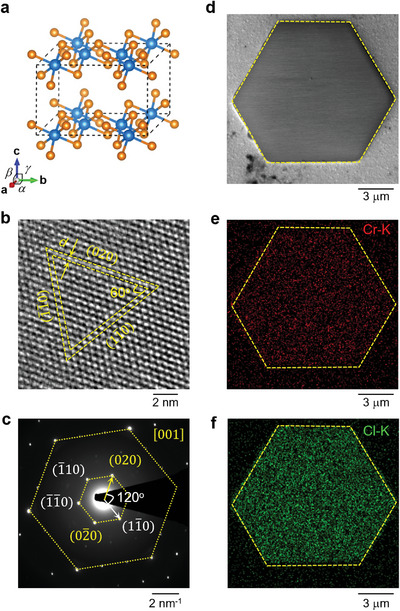
Structural characterization and element analysis of CrCl_3_ flakes. a) Schematic unit cell of monoclinic CrCl_3_, with *a* = 5.959 Å, *b* = 10.321 Å, *c* = 6.114 Å, *α* = *γ* = 90^o^, and *β* = 108.49°. b) HRTEM micrograph and c) SAD pattern taken on a thick CrCl_3_ flake along [001] zone axis. d) SEM image of a thick CrCl_3_ flake, with element mapping of e) Cr and f) Cl.

We carry out TEM, SEM, and Raman measurements to characterize the sample structure and stoichiometry. Our studies show that CrCl_3_ can be easily damaged when exposed to electron beam and laser excitation (Figure S3, Supporting Information). To ensure the data quality, we have reduced the exposure time and used minimal laser power in these measurements and focused on characterizing relatively thick samples (>20 nm). At room temperature, CrCl_3_ possesses the monoclinic structure, which belongs to the C2/m space group (**Figure** **2**a).^[^ ^28^ ^]^ The Cr atoms form a honeycomb structure in the *a*‐*b* plane, with each Cr atom surrounded by the Cl octahedron. Figure 2b shows an HRTEM image of CrCl_3_, where the crystalline planes of (020), ($1\bar{1}0$), ($\bar{1}\bar{1}0$) make a quasi‐equilateral triangle. The inter‐planar spacing *d* is about 5.1 Å, agreeing with the expected lattice parameter for the monoclinic structure. The corresponding selected area diffraction (SAD) pattern (Figure 2c) is also consistent with the monoclinic crystal structure.^[^ ^28^ ^]^ The sharp diffraction peaks and the absence of impurity phases confirm the high crystallinity of the sample.

The stoichiometry of the sample is investigated using SEM‐EDS (Figure 2d–f). Element mapping of the Cr K‐line (Figure 2e) and Cl K‐line (Figure 2f) reveals a homogeneous distribution. From the EDS spectrum, we extract a Cr/Cl ratio of 0.304 (Figure S4, Supporting Information), reasonably close to the ideal ratio of 1/3 considering the uncertainties related to EDS quantitative analysis, as there is significant background signal from the underlying substrate for thin film samples.^[^ ^22^ ^]^ No signal of sulfur is detected in the EDS spectrum, confirming that the sample purity is not affected by the S powder used for promoting sample nucleation.

**Figure 3:**
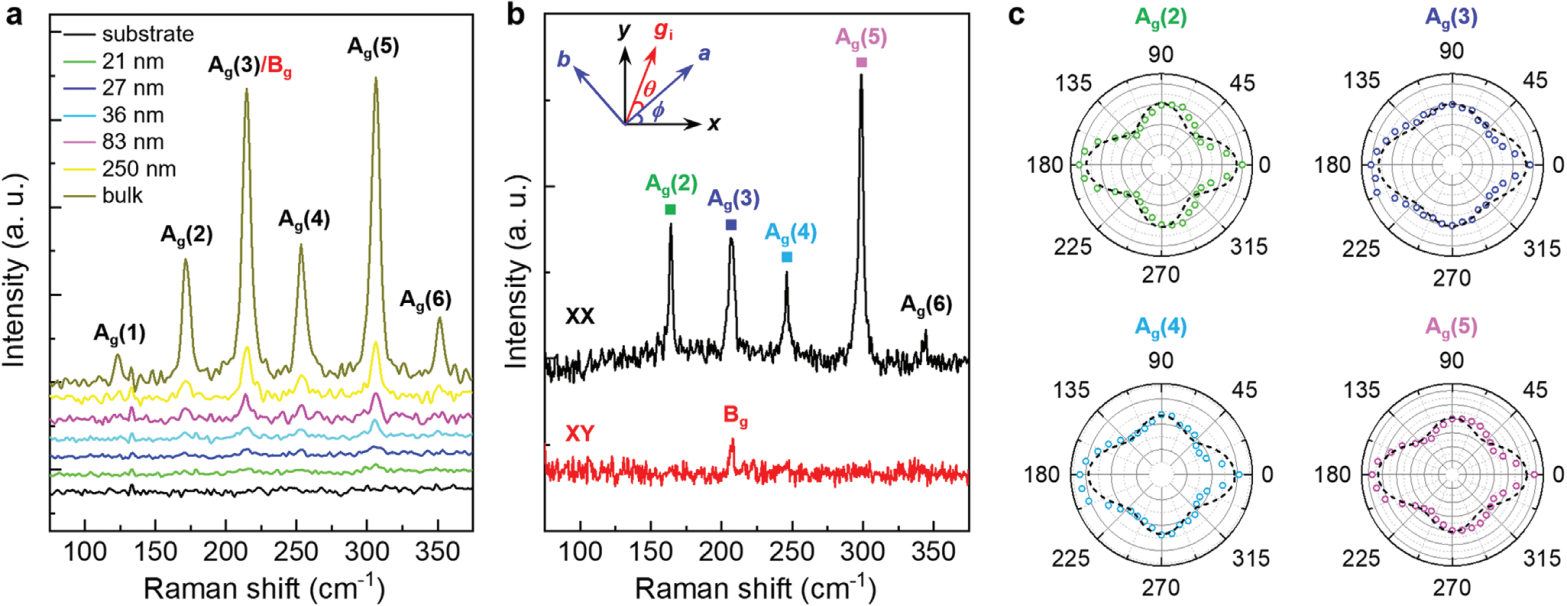
Raman characterizaiton of CrCl_3_ flakes. a) Raman spectra of CrCl_3_ flakes with different thicknesses. b) Raman spectra of a 43 nm CrCl_3_ flake measured in parallel (XX) and perpendicular (XY) configurations. Inset: Schematic of crystalline orientations and laboratory coordinates, where the relative angle *ϕ* between *x*‐axis and sample *a*‐axis is arbitrary. c) Polar plots of integrated Raman intensity for different A_g_ modes of the same CrCl_3_ flake shown in (b).

Next, we carry out Raman studies at room temperature. To minimize the sample damage by laser heating,^[^ ^29^ ^]^ we transfer the samples onto Au‐coated SiO_2_/Si substrates to facilitate energy dissipation. **Figure** **3**a shows the Raman spectra of CrCl_3_ flakes with different thicknesses. For micron‐thick bulk samples, we observed six Raman peaks at about 123, 165, 207, 244, 300, and 344 cm^−1^, which are denoted as A_g_(1), A_g_(2), A_g_(3)/B_g_, A_g_(4), A_g_(5), and A_g_(6) modes, respectively. The spectrum agrees with the monoclinic structure of CrCl_3_.^[^ ^3^, ^20^ ^]^ The Raman signal decreases with sample thickness and becomes hard to resolve in flakes thinner than 20 nm. For the signal that can be detected, there is no noticeable peak shift with flake thickness.

Figure 3b shows the polarized Raman spectra taken on a 43 nm CrCl_3_ flake. Compared with the polarized Raman spectra of bulk CrCl_3_ crystal,^[^ ^30^ ^]^ we only resolve five A_g_ phonon modes in the parallel polarization (XX) and one B_g_ mode in the perpendicular polarization (XY) due to the relatively low signal strength in thin flakes. The peak position for the B_g_ mode (about 207 cm^−1^) contains two modes B_g_(3/4) with degenerate energy.^[^ ^30^ ^]^ Figure 3c shows the polar maps of XX Raman intensity for the four A_g_ modes with relatively high intensity, where the angle of the incident light polarization *θ* is defined with respect to the *a*‐axis of CrCl_3_ (Figure S5, Supporting Information). All A_g_ modes exhibit twofold symmetry, with four local maxima occurring at *θ* = 0°, 90°, 180°, 270°. The intensity at 0° and 180° is higher than that at 90° and 270°. The Raman intensity is proportional to $|g_{s}\tilde{R}g_{i}^{T}{|}^{2}$, where ***g***_i_ (***g***_s_) is the polarization vector of the incident (scattered) light and $\tilde{R}$ is the Raman tensor.^[^ ^31^ ^]^ In the XX configuration, ***g***_s_ = ***g***_i_∝(cos*θ*, sin*θ*, 0). For a monoclinic structure, the angular‐dependent Raman response in XX is given by: *I*(A_g_)∝|*a*cos^2^ *θ* + *b*sin^2^ *θ*|^2^ and *I*(B_g_)∝*e* ^2^sin^2^(2*θ*), where *a*, *b*, and *e* are fitting parameters.^[^ ^31^ ^]^ Previous studies have shown that both A_g_ and B_g_ modes can contribute to the polar mapping,^[^ ^31^ ^]^ so the overall Raman intensity can be expressed as:

(1) $$I^{\prime }\propto {\left|a\cos^{2}\theta +b\sin^{2}\theta \right|}^{2}+e^{2}\sin^{2}\left(2\theta \right)$$

As shown in Figure 3c, Equation (1) well describes the angular dependence of Raman intensity, further confirming that CrCl_3_ is crystallized in the monoclinic structure.

### 2.3 Characterization of Few‐Layer CrCl_3_ Tunnel Junctions

**Figure 4:**
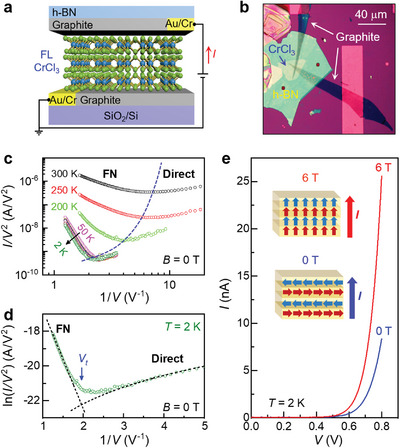
Tunneling characteristic of a graphite/6‐layer CrCl_3_/graphite device encapsulated with h‐BN. a) Device schematic. b) Optical image. c) Zero field *I*/*V* ^2^ versus 1/*V* at 300, 250, 200, 50, 25, 22, 20, 18, 12, and 2 K. The dashed line serves as a guide to the eye. d) Zero field ln(*I*/*V* ^2^) versus 1/*V* at 2 K with fits to Equations (2) and (3) (dashed lines). e) Tunneling *I*–*V* at 2 K with *B*_⊥_ = 0 and 6 T. Inset: Schematic of spin orientation in CrCl_3_ with and without magnetic field.

To probe the magnetic properties of the sample, we fabricate few‐layer CrCl_3_ into tunnel junction devices (**Figure** **4**a) and characterize their tunneling magnetoresistance (TMR). Figure 4b shows a device composed of a 6‐layer CrCl_3_ tunnel barrier (Figures S6 and S7, Supporting Information) sandwiched between top and bottom thin graphite flakes transferred on a SiO_2_ substrate with prepatterned gold electrodes (Experimental Section). The effective area of the tunnel junction is about 10.8 µm^2^. The device is then encapsulated by a top h‐NB flake to avoid ambient degradation. At room temperature, the *I*–*V* characteristic of the device is highly stable for over 2 months in the ambient conditions, which is the duration of measurement (Figure S8, Supporting Information).

Figure 4c shows the tunneling characteristic of the device at various temperatures. The tunneling current decreases rapidly with decreasing temperature below 300 K and exhibits weak temperature‐dependence below 50 K. Plotting *I*/*V* ^2^ versus 1/*V* reveals two distinct regimes, which can be understood by considering the evolution of the dominating tunneling mechanism. At low bias *V* <<*Φ*/*e*, where *Φ* is the tunnel barrier height and *e* is the elementary charge, the tunneling current is dominated by the direct tunneling mechanism, with the tunneling current given by:^[^ ^32^, ^33^ ^]^

(2) $$I\propto Ve^{\left(-\frac{2d\sqrt{2m^{*}\Phi }\;}{\hbar }\right)}$$

Here m^*^ is the effective mass, *ħ* is the reduced Plank constant, and *d* is the thickness of the CrCl_3_ flake. At *V* > *Φ*/*e*, the Fowler–Nordheim (FN) mechanism becomes dominant, and the current can be expressed as:^[^ ^32^, ^33^ ^]^

(3) $$I\propto V^{2}e^{\left(-\frac{4d\sqrt{2m^{*}\Phi^{3}}\;}{3\hbar eV}\right)}$$

Equations (2) and (3) can well capture the data shown in Figure 4c. The transition voltage between these two regimes decreases with increasing temperature, illustrating the enhanced contribution of thermo‐carriers tunneling through the bias‐modified tunnel barrier.^[^ ^34^ ^]^

We then use the transition between the direct and FN tunneling regimes at low temperature to estimate the tunnel barrier height Φ.^[^ ^32^, ^35^ ^]^ In Figure 4d, we plot $\ln(\frac{I}{V^{2}})$ versus 1/*V* at 2 K and superimpose the fitting curves for the direct tunneling regime, i.e., $\ln\left(\frac{I}{V^{2}}\right)\propto \ln\left(\frac{1}{V}\right)$ (Equation (2)), and the FN regime, i.e., $\ln\left(\frac{I}{V^{2}}\right)\propto \frac{1}{V}$ (Equation (3)). The transition voltage *V*_t_ is defined as the crossing point of these two behaviors (*V*_t_ ≈ 0.51 V), which has been used to estimate the height of the tunnel barrier. As the transition is relatively broad, this can lead to about 10% uncertainty in the estimated Φ. Assuming *Φ* = *eV*_t_ = 0.51 eV and considering the layer number of the flake to be 6 ± 1 (Figure S7, Supporting Information), we extract the effective mass for the CrCl_3_ tunnel barrier to be m* = (0.5 ± 0.1)*m*_0_, where *m*_0_ is the free electron mass.^[^ ^36^ ^]^

Figure 4e shows the tunneling *I*–*V* relation at 2 K with and without a perpendicular magnetic field *B*_⊥_. Applying a magnetic field increases the tunneling current, which can be attributed to spin alignment in CrCl_3_ induced by the magnetic field. Without the magnetic field, the spins in the adjacent layers are antiparallel to each other, which suppresses the electron tunneling probability, yielding an effectively higher tunnel barrier height. An applied field of 6 T can align the spins of all layers along the out‐of‐plane direction, resulting in higher *I*. At *V* = 0.8 V, the tunneling current changes from 8.4 nA at 0 T to 25.6 nA at 6 T, corresponding to a TMR (6 T) = $100\%\;\times \frac{I(6\;T)-\;I(0\;T)}{I(0\;T)}$ = 206%, which is significantly higher than that obtained on bilayer and trilayer CrCl_3_ tunneling devices at this temperature in previous experiments.^[^ ^5^ ^]^ The enhanced TMR shows that the spin filtering efficiency increases with increasing tunnel barrier thickness.^[^ ^2^ ^]^

**Figure 5:**
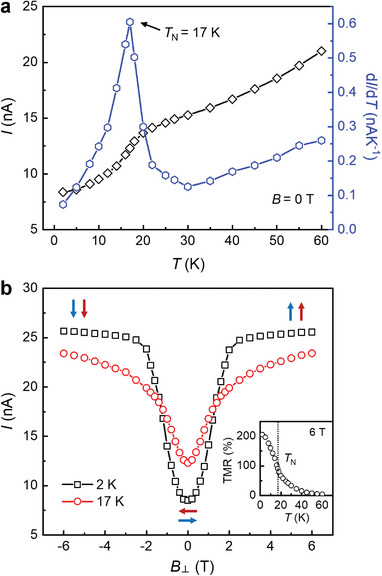
Tunneling magnetotransport of the 6‐layer CrCl_3_ tunnel junction device. a) Temperature‐dependent tunneling current at zero magnetic field. b) Tunneling current versus *B*_⊥_ at 2 and 17 K. *V* = 0.8 V. Inset: TMR ratio versus *T* at *B*_⊥_ = 6 T. The dotted line marks *T*_N_.

From the temperature‐dependence of zero field tunneling current and its derivative *dI/dT* (**Figure** **5**a), we identify a clear kink at 17 K, which corresponds to the *T*_N_. The *T*_N_ value is consistent with previous reports of bulk^[^ ^21^ ^]^ and exfoliated CrCl_3_.^[^ ^1^, ^2^, ^4^, ^5^ ^]^ Below and above *T*_N_, the tunneling current exhibits distinct magnetic field dependence. As shown in Figure 5b, at 2 K, *I* rises rapidly with increasing magnetic field and saturates at around *B*_⊥_ = 2.5 T. Below *T*_N_, the magnetic field aligns the in‐plane, anti‐aligned interlayer spins to the out‐of‐plane orientation, which yields higher tunneling current.^[^ ^1^, ^2^, ^4^, ^5^ ^]^ Once the spins are fully aligned, increasing the magnetic field no longer changes the tunneling current. At 17 K, in contrast, the tunneling current exhibits a weaker magnetic field dependence and does not saturate in field up to 6 T. Above *T*_N_, the spins do not have long‐range order and are randomly oriented. The magnetic field is thus not sufficient to fully align the spins. This change is also reflected in the temperature‐dependence of TMR at 6 T (Figure 5b inset), which decreases monotonically with increasing temperature and exhibits a deflection point around *T*_N_. We also note that the change of tunneling current below *T*_N_ is gradual, in contrast to the sharp change observed in CrI_3_.^[^ ^6^ ^]^ This is consistent with the weak in‐plane magnetic anisotropy for CrCl_3_, where the out‐of‐plane magnetic field induces continuous spin rotation rather than directly flipping the spin orientation.^[^ ^1^, ^2^, ^4^, ^5^, ^21^ ^]^

## 3 Conclusion

In conclusion, we have successfully synthesized large CrCl_3_ flakes down to monolayer thickness using the physical vapor transport technique, with high crystallinity and homogeneous chemical composition achieved. With h‐BN encapsulation, few‐layer CrCl_3_‐based tunneling devices exhibit high ambient stability for more than 2 months. The tunneling magnetoresistance reveals that few‐layer CrCl_3_ flakes possess a Néel temperature of 17 K, in‐plane magnetic anisotropy, and tunneling magnetoresistance of >200% below *T*_N_. Our study enables the direct growth of large size atomically thin CrCl_3_ flakes, paving the path for implementing this material for scalable 2D spintronic applications.

## 4 Experimental Section

### Synthesis

High‐quality 2D vdW CrCl_3_ flakes were deposited in a horizontal single‐zone furnace (Thermo Scientific TF55035‐A1) with a 1 inch diameter quartz tube by the PVT technique. A quartz boat with CrCl_3_ source powder (99.99%, Alfa Aesar) was placed at the center of the single‐zone tube furnace. A small amount of sulfur powder (99.9995%, Alfa Aesar) was loaded in the upstream of the tube to facilitate sample nucleation. The substrate was placed in the tube at about 10 cm downstream from the CrCl_3_ source powder. Three types of substrates, mica (highest grade V1 mica disc, MIT), HOPG (Grade 3, SPI), and 300 nm SiO_2_/Si were investigated. Before growth, the system was purged by Ar gas three times. During sample growth, the furnace was heated up to 700–750 °C at a rate of 30 °C min^−1^ with 40 standard cubic centimeters per minute (sccm) Ar process gas, and the tube was kept at one atmosphere pressure. After 5 min growth, the furnace was cooled down to room temperature naturally.

### Sample Characterizations

The thickness and surface morphology of as‐grown CrCl_3_ flakes were characterized by AFM (Bruker Multimode 8) with the tapping mode. SEM was performed using an FEI Helios Nanolab 660 with a field emission gun at 2 kV. The chemical element analysis was conducted by EDS using the point and mapping modes in SEM. HRTEM studies were performed in an FEI Tecnai Osiris electron microscope operated at 200 kV. Nonpolarized Raman spectra were collected by a Thermo Scientific DXR Raman microscope with a 532 nm laser, a 100× objective, exposure time of 30 s, 0.2 mW laser power, and a 900 lines mm^−1^ grating. Polarized Raman spectra were recorded using a Harina/Princeton Acton 7500i/spectrometers equipped with a 532 nm laser, with a 50× objective, 0.2 mW incident laser power, integration time of 20 min, and 1800 lines mm^−1^ grating. The excitation laser and collected Raman signal were collinearly polarized. For the angular dependence measurements, the angle step was 5° for a half‐wave plate, which was 10° in the polar map. For SEM, TEM, and Raman characterizations, the CrCl_3_ flakes were transferred onto Au‐coated (10 nm) SiO_2_/Si substrates (SEM and Raman) and TEM chips (Silicon Nitride Support Film, 50 nm with 0.5 × 0.5 mm Window) using the all‐dry stamping transfer technique.

### Device Fabrication and Electrical Characterizations

Au/Cr (20/5 nm) electrodes were prepatterned into two‐point geometry on SiO_2_/Si substrates using photolithography followed by e‐beam evaporation. The tunnel junction devices were assembled by the all‐dry stamping transfer method, which was performed on an optical microscope equipped with a stamping stage. The as‐grown CrCl_3_ flakes were picked up from the mica substrate by an elastomeric film (Gel‐Film WF × 4 1.5 mil from GelPak), which was adhered to a glass slide fixed on the stamping stage. The thin graphite electrodes and the h‐BN protection layer were mechanically exfoliated. The graphite, few‐layer CrCl_3_, and h‐BN flakes were picked up sequentially by gel‐films and stacked into h‐BN encapsulated graphite/CrCl_3_/graphite heterostructures on top of the prepatterned SiO_2_/Si substrates (Section S6, Supporting Information). The electrical measurements were carried out in a Quantum Design PPMS using an external Keysight 1500A Semiconductor Device Parameter Analyzer.

## Conflict of Interest

The authors declare no conflict of interest.

## Author Contributions

X.H. conceived the project. X.H. and J.W. designed the experiments. J.W. carried out sample deposition, AFM and SEM characterizations, device fabrication, and magnetotransport studies. Z.A. and J.E.S. performed the TEM studies. J.W., D.L., J.C., and X.L. conducted the Raman studies. T.T. and K. W. contributed to the h‐BN samples. J.W. and X.H. wrote the manuscript. All authors discussed the results and contributed to the manuscript preparation.

## Supporting information

Supporting Information

## Data Availability

The data that support the findings of this study are available in the supplementary material of this article.
