# Supplementary material for: Physical Vapor Transport Growth of Antiferromagnetic CrCl3 Flakes Down to Monolayer Thickness
Source: Adv Sci (Weinh). 2022 Dec 1;10(3):2203548. doi: 10.1002/advs.202203548 (PMC9875658; doi:10.1002/advs.202203548)
Supplement: Supplementary file 1 — Supporting Information [file ADVS-10-2203548-s001.pdf]

Supporting Information

Physical Vapor Transport Growth of Antiferromagnetic $CrCl_3$ Flakes Down to Monolayer Thickness

*Jia Wang, Zahra Ahmadi, David Lujan, Jeongheon Choe, Takashi Taniguchi, Kenji Watanabe, Xiaoqin Li, Jeffrey E. Shield, and Xia Hong**

Jia Wang, Prof. Xia Hong*
Department of Physics and Astronomy & Nebraska Center for Materials and Nanoscience, University of Nebraska-Lincoln, Lincoln, NE 68588-0299, USA
E-mail: xia.hong@unl.edu

Dr. Zahra Ahmadi, Prof. Jeffrey E. Shield
Department of Mechanical and Materials Engineering, University of Nebraska-Lincoln
Lincoln, NE 68588-2526, USA

David Lujan, Jeongheon Choe, Prof. Xiaoqin Li
Department of Physics, University of Texas at Austin, Austin, TX 78712-1192, USA

Dr. Takashi Taniguchi
International Center for Materials Nanoarchitectonics, National Institute for Materials Science, 1-1 Namiki, Tsukuba 305-0044, Japan

Dr. Kenji Watanabe
Research Center for Functional Materials, National Institute for Materials Science, 1-1 Namiki, Tsukuba 305-0044, Japan

Contents:

1. Thickness Distribution of $CrCl_3$ Samples
2. Room Temperature Stability of $CrCl_3$ Flakes
3. Sample Damage upon TEM and Raman Measurements
4. Element Analysis
5. Polarized Raman Analysis of Crystalline Orientation
6. Assembly of h-BN Encapsulated Graphite/$CrCl_3$/Graphite Tunnel Junction
7. Thickness of $CrCl_3$ Tunnel Barrier
8. Ambient Stability of h-BN Encapsulated Few-Layer $CrCl_3$ Tunnel Junction

1. Thickness Distribution of $CrCl_3$ Samples

Figure S1a shows an optical image of isolated $CrCl_3$ flakes on a mica substrate. It is challenging to identify the ultrathin flakes due to the poor optical contrast for $CrCl_3$ on mica. We have performed systematic atomic force microscopy (AFM) imaging to search for ultrathin flakes over this area and identified 8 ultrathin samples, including monolayer (1L), bilayer (2L), and trilayer (3L) flakes, out of a total of 30 flakes (Figure S1a-b), corresponding to about 25% yield. Figure S1c shows an optical image of a long stripe sample with over 1 mm length. Based on the AFM measurements (Figure S1d), we estimate that about 60% of the stripe sample is 3L thick.

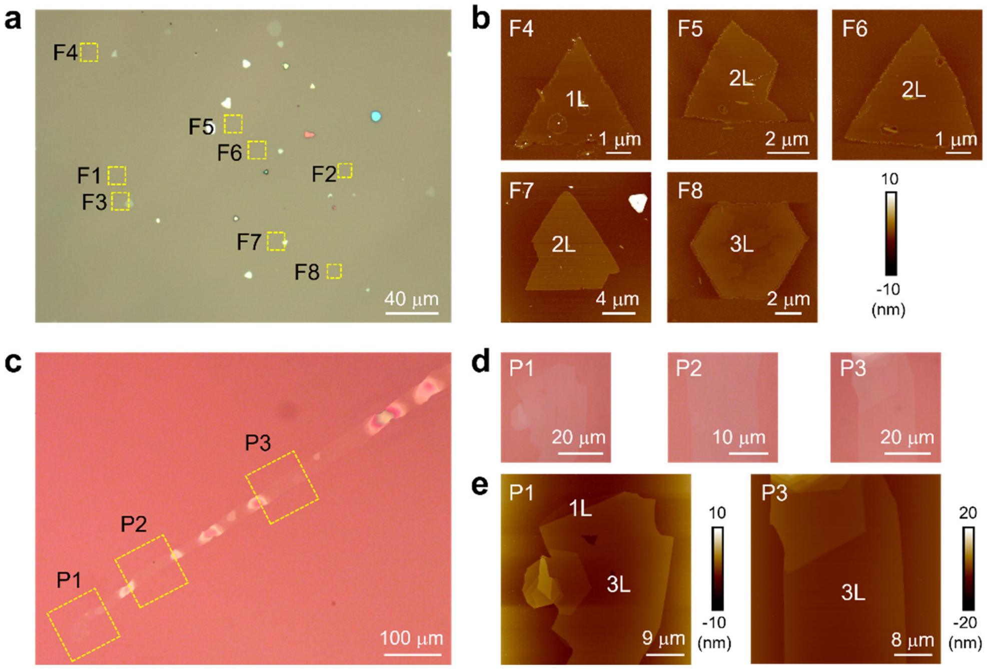

Figure S1. Thickness distribution of $CrCl_3$ samples. a) Optical image of as-grown $CrCl_3$ flakes on mica. The AFM images of flakes F1-F3 are shown in Figure 1h, Figure 1g, and Figure S2c, respectively. b) AFM images of $CrCl_3$ flakes F4-F8 marked in (a) (dashed boxes). c) Optical image of a long stripe $CrCl_3$ sample on mica. d) Expanded optical images of areas P1-P3 marked in (c) (dashed boxes). e) AFM images taken on the areas P1 and P3. The AFM image of P2 is shown in Figure 1i in the main text.

2. Room Temperature Stability of $CrCl_3$ Flakes

Figure S2 shows the AFM images of $CrCl_3$ flakes with various thicknesses taken at different time. The stability of the flakes is discussed in Section 2.1 in the main text.

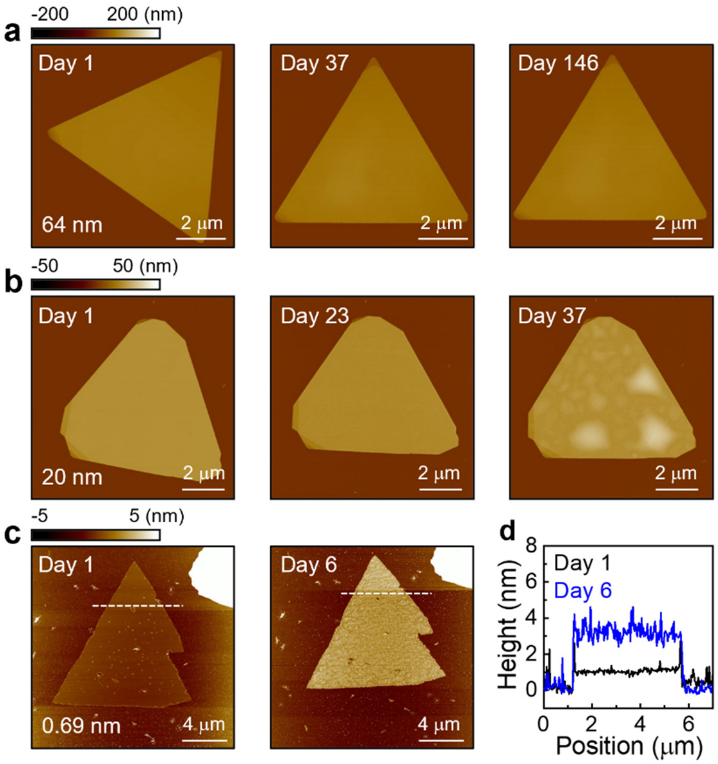

Figure S2. Room temperature stability of $CrCl_3$ flakes. a) AFM images of a 64 nm thick $CrCl_3$ flake on mica taken on Day 1, 37, 146 after sample growth. b) AFM images of a 20 nm thick $CrCl_3$ flake on mica taken on Day 1, 23, and 37 after sample growth. c) AFM images of a 1L $CrCl_3$ flake on mica taken on Day 1 and 6 after sample growth, with d) the corresponding height profiles along the dashed lines. This sample is the flake F3 marked in Figure S1a.

3. Sample Damage upon TEM and Raman Measurements

The $CrCl_3$ samples can be easily damaged upon exposure to high-energy electron beam and high-power laser excitation. **Figure S3** shows the sample images after TEM and Raman measurements, where the damaged spots are clearly visible. To ensure the collected data quality being preserved during measurements, we have reduced the exposure time and used minimal laser power, which result in very weak signals from thin flakes. For example, as shown in Figure 3a, the Raman response for flakes thinner than 20 nm cannot be barely resolved at the laser power of 0.2 mW. Therefore, our TEM and Raman characterizations are focused on relatively thick flakes.

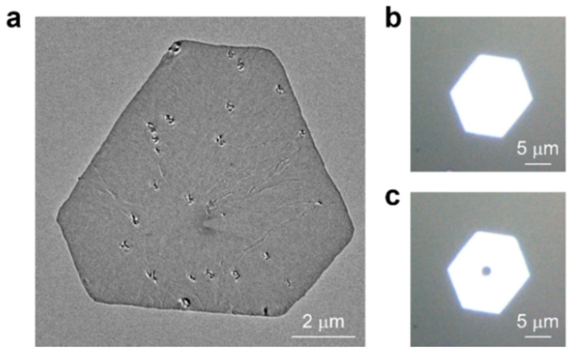

Figure S3. Sample Damage upon TEM and Raman Measurements. a) TEM image of a $CrCl_3$ flake showing damaged spots after electron beam exposure. b-c) Optical images of a $CrCl_3$ flake before (b) and after (c) Raman measurements.

4. Element Analysis

Figure S4 shows the energy dispersive x-ray spectroscopy (EDS) characterization of the flake discussed in Figures 2d-f in the main text. The highest peaks are associated with Si and Au, which come from the Au coated Si substrate. In the expanded spectrum, we show the major Cr and Cl peaks, from which we analyze the atomic percentage of the elements.

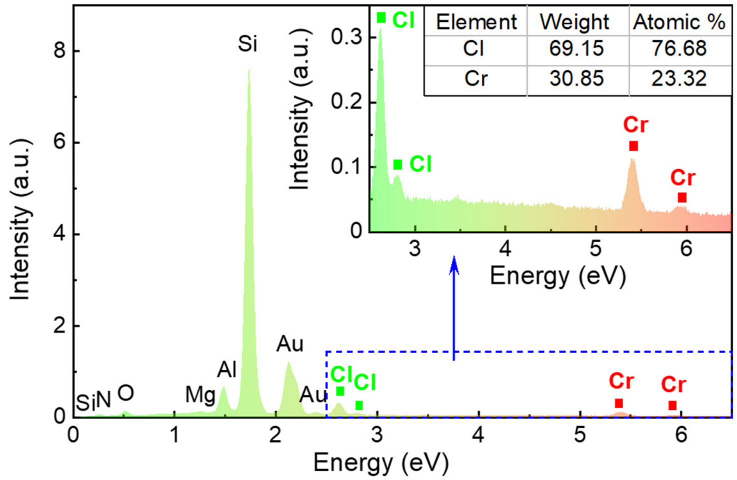

Figure S4. EDS spectrum of a 150 nm thick $CrCl_3$ flake. Inset: Expanded view showing the Cl and Cr peaks.

5. Polarized Raman Analysis of Crystalline Orientation

As discussed in Figure 3c, the Raman intensity of the A_g modes reaches maximum in parallel polarization when $\theta = 0°$, namely, $\boldsymbol{g}_S \parallel \boldsymbol{a}$ (**Figure S5**a). Based on the Raman polar mapping, we

can identify the crystalline orientation of the sample. Figure S5b shows the assigned crystal axes and facets of the $CrCl_3$ flake discussed in Figures 3b-c. It confirms that the {020} and {110} facets are favored during nucleation and growth, which agrees with our TEM analysis (Figures 2b-c).

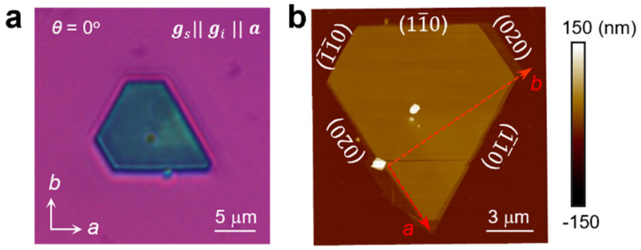

Figure S5. Crystalline orientation of $CrCl_3$ flake. a) Optical and b) AFM images of the 43 nm flake discussed in Figures 3b-c, with the crystalline orientation labelled.

6. Assembly of h-BN Encapsulated Graphite/$CrCl_3$/Graphite Tunnel Junction

Figure S6 shows the optical images of the step-by-step process flow for assembling the h-BN encapsulated graphite/few-layer $CrCl_3$/graphite tunnel junction.

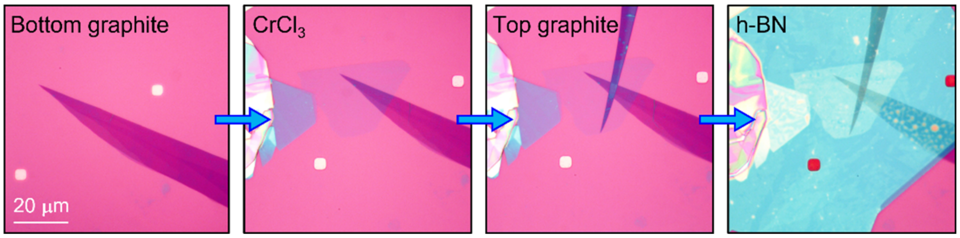

Figure S6. Optical images showing the process flow for assembling the h-BN encapsulated graphite/$CrCl_3$/graphite tunnel junction.

7. Thickness of $CrCl_3$ Tunnel Barrier

Figure S7a shows the optical image of the assembled few-layer $CrCl_3$ tunnel junction device discussed in Figures 4-5 before encapsulation with h-BN. The thickness of the $CrCl_3$ flake has been estimated using calibrated color contrast for $CrCl_3$ on SiO_2. As shown in Figure S7b, the color contrast of the tunnel barrier suggests that it is thicker than flake F1 (4-layer) and thinner than flake F2 (8-layer), whose thicknesses are calibrated by AFM (Figure S7b-e). We thus conclude that the thickness of the tunnel barrier is 6±1 layer.

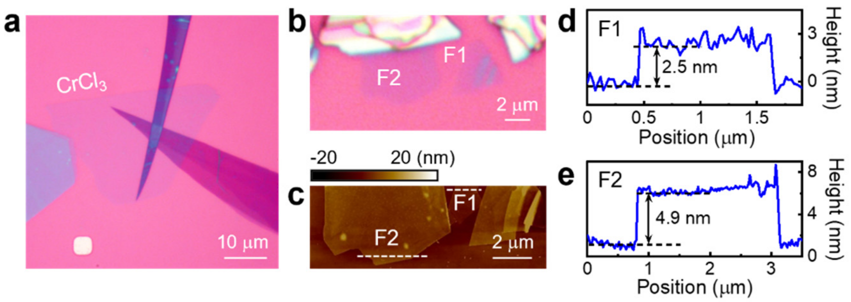

Figure S7. Thickness calibration for the $CrCl_3$ tunnel barrier. a) Optical image of the graphite/few-layer $CrCl_3$/graphite device before h-BN encapsulation. b-e) Characterization of few-layer $CrCl_3$ flakes on bare SiO_2/Si subsrate for thickness calibration. b) Optical image. c) AFM image, with the height profiles along the dahsed lines for flakes F1 (d) and F2 (e).

8. Ambient Stability of h-BN Encapsulated Few-Layer $CrCl_3$ Tunnel Junction

Figure S8 shows the tunneling *I-V* of the 6-layer $CrCl_3$ tunnel junction device at 300 K taken at various time. At room temperature, there is no prominent change in the *I-V* characteristic, showing excellent ambient stability for over 2 months for the h-BN encapsulated device. This result is discussed in Section 2.3 in the main text.

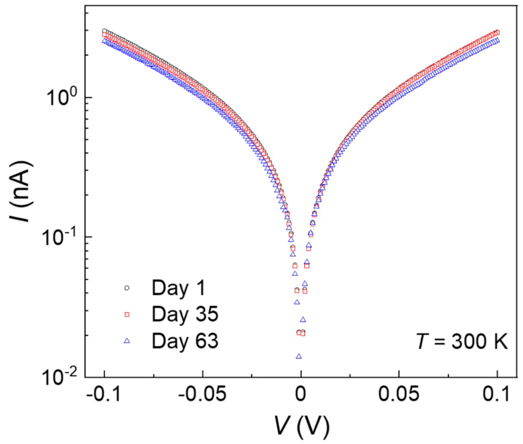

Figure S8. Tunneling *I-V* of the 6-layer $CrCl_3$ tunnel junction device at 300 K taken on Day 1, 35, and 63 after fabrication.
